# (*E*)-1,1′-Bis[(*E*)-but-2-en­yl]-3,3′-(propane-1,3-di­yl)bis­(1*H*-benzimidazol-3-ium) dibromide monohydrate

**DOI:** 10.1107/S160053680803095X

**Published:** 2008-10-18

**Authors:** Mehmet Akkurt, Sema Öztürk Yıldırım, Nihat Şireci, Hasan Küçükbay, Orhan Büyükgüngör

**Affiliations:** a Department of Physics, Faculty of Arts and Sciences, Erciyes University, 38039 Kayseri, Turkey; b Department of Chemistry, Faculty of Arts and Sciences, Adıyaman University, Adıyaman, Turkey; c Department of Chemistry, Faculty of Arts and Sciences, Ínönü University, 44280 Malatya, Turkey; d Department of Physics, Faculty of Arts and Sciences, Ondokuz Mayıs University, 55139 Samsun, Turkey

## Abstract

The title compound, C_25_H_30_N_4_^2+^·2Br^−^·H_2_O, was synthesized from 1,1′-propyl­enedibenzimidazole and (*E*)-1-bromo­but-2-ene in dimethyl­formamide solution. The two benzimidazole ring systems are essentially planar, with maximum deviations of 0.011 (4) and 0.023 (3) Å. The dihedral angle between these two ring systems is 25.87 (15)°. The crystal structure is stabilized by inter­molecular O—H⋯Br and C—H⋯Br hydrogen-bonding inter­actions. Atmospheric water was incorporated into the crystal structure.

## Related literature

For bond-length data, see: Allen *et al.* (1987 ▶). For general background, see: Sakai *et al.* (1989 ▶); Tidwell *et al.* (1993 ▶); Küçükbay *et al.* (1995 ▶, 2001 ▶); Turner & Denny (1996 ▶); Hall *et al.* (1998 ▶). For related structures, see, for example: Öztürk *et al.* (2003 ▶); Akkurt *et al.* (2003 ▶, 2006 ▶).
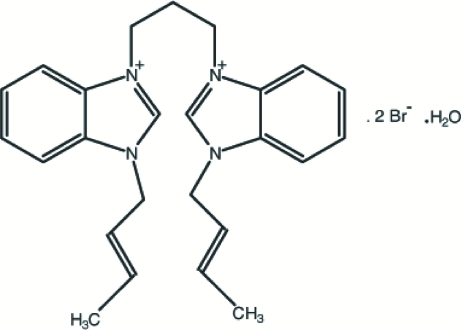


## Experimental

### 

#### Crystal data

C_25_H_30_N_4_^2+^·2Br^−^·H_2_O
*M* *_r_* = 564.35
Triclinic, 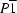

*a* = 8.7989 (9) Å
*b* = 11.1878 (13) Å
*c* = 14.8813 (14) Å
α = 106.381 (8)°
β = 96.490 (8)°
γ = 106.227 (8)°
*V* = 1319.9 (3) Å^3^
*Z* = 2
Mo *K*α radiation
μ = 3.09 mm^−1^
*T* = 296 K
0.53 × 0.45 × 0.31 mm

#### Data collection

Stoe IPDSII diffractometer
Absorption correction: integration (*X-RED32*; Stoe & Cie, 2002 ▶) *T*_min_ = 0.291, *T*_max_ = 0.447
16109 measured reflections
6181 independent reflections
4101 reflections with *I* > 2σ(*I*)
*R*_int_ = 0.063

#### Refinement

*R*[*F* ^2^ > 2σ(*F* ^2^)] = 0.054
*wR*(*F* ^2^) = 0.124
*S* = 1.05
6181 reflections
297 parameters
H atoms treated by a mixture of independent and constrained refinement
Δρ_max_ = 0.56 e Å^−3^
Δρ_min_ = −0.41 e Å^−3^

### 

Data collection: *X-AREA* (Stoe & Cie, 2002 ▶); cell refinement: *X-AREA*; data reduction: *X-RED32* (Stoe & Cie, 2002 ▶); program(s) used to solve structure: *SIR97* (Altomare *et al.*, 1999 ▶); program(s) used to refine structure: *SHELXL97* (Sheldrick, 2008 ▶); molecular graphics: *ORTEP-3 for Windows* (Farrugia, 1997 ▶); software used to prepare material for publication: *WinGX* (Farrugia, 1999 ▶).

## Supplementary Material

Crystal structure: contains datablocks global, I. DOI: 10.1107/S160053680803095X/sj2540sup1.cif

Structure factors: contains datablocks I. DOI: 10.1107/S160053680803095X/sj2540Isup2.hkl

Additional supplementary materials:  crystallographic information; 3D view; checkCIF report

## Figures and Tables

**Table 1:** Hydrogen-bond geometry (Å, °)

*D*—H⋯*A*	*D*—H	H⋯*A*	*D*⋯*A*	*D*—H⋯*A*
O1—H*W*1⋯Br1	0.81 (8)	2.54 (8)	3.342 (6)	171 (8)
O1—H*W*2⋯Br2^i^	0.89 (9)	2.45 (8)	3.333 (5)	171 (7)
C4—H4*A*⋯Br2^ii^	0.97	2.81	3.773 (4)	172
C4—H4*B*⋯Br2^i^	0.97	2.86	3.802 (6)	164
C11—H11⋯Br1	0.93	2.76	3.536 (4)	142
C12—H12*A*⋯Br2	0.97	2.89	3.822 (5)	161
C19—H19⋯Br2^iii^	0.93	2.92	3.816 (5)	162
C21—H21⋯Br1	0.93	2.65	3.550 (5)	162

Symmetry codes: (i) 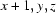; (ii) 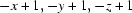; (iii) 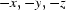.

**Fig. 1.**
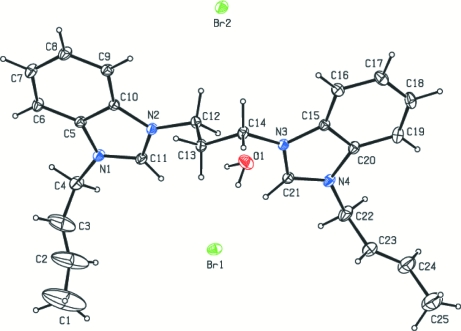
An ORTEP view of (I), with the atom-numbering scheme and 10% probability displacement ellipsoids.

**Fig. 2.**
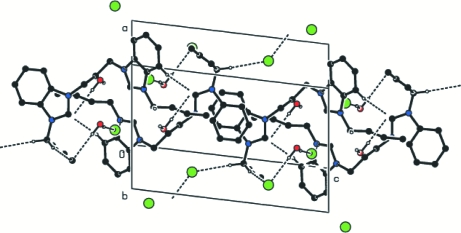
The packing and hydrogen bonding of (I), viewed down the b axis. Hydrogen bonds are drawn as dashed lines and H atoms not involved in these interactions have been omitted for clarity.

## supplementary crystallographic information

### Comment

In the light of the general importance of benzimidazole compounds, the study of bisbenzimidazoles and their related derivatives has remained an active area of research despite extensive investigation. They are present in various naturally occurring drugs such as omeprazole, astemizole and emedastine difumarate (Sakai *et al.*, 1989). Substituted benzimidazole compounds are established pharmacophores in parasitic chemotheraphy. They also show antiviral (Tidwell *et al.*, 1993), antimicrobial (Küçükbay *et al.*, 1995; Küçükbay *et al.*, 2001), antitumor (Turner and Denny, 1996), antihistaminic, anticoagulant and anti-inflammatory activities (Hall *et al.*, 1998). The objective of this study was to elucidate the crystal structure of the title compound (I) and to compare the results obtained with our previous studies on related bis-benzimidazole compounds (Öztürk *et al.*, 2003; Akkurt *et al.*, 2003; Akkurt *et al.*, 2006).

In (I) (Fig. 1), the bond lengths and angles are generally within normal ranges (Allen *et al.*, 1987). The two benzimidazole ring systems of (I), A (N1/N2/C5–C11) and B (N3/N4/C15–C21), are essentially planar, with maximum deviations of 0.011 (4) Å for C10 and -0.10 (5) Å for C11 in A, and 0.023 (3) Å for N3 and 0.016 (5) Å for C19 in B. The dihedral angle between these two ring systems A and B is 25.87 (15) °. This angle is 31.84 (11)° in 3,30-bis(cyclohexylmethyl)- 1,10-propylenedibenzimidazolium dibromide monohydrate (Akkurt *et al.*, 2006) and 88.42 (4)° in 3,30-bis(3- cyanopropyl)-1,10-propylene- di(benzimidazolium) dichloride dihydrate (Akkurt *et al.*, 2003). This divergence may be due to the interactions of the different substituents bounded to the benzimidazole ring system.

The crystal structure of (I) is stabilized by inter and intramolecular O—H···Br and C—H···Br hydrogen bonding interactions, involving the H atoms of the water molecule (Table 1 and Fig. 2).

### Experimental

A mixture of 1,1'-propylenedibenzimidazole (0.9 g, 3.26 mmol) and (E)-1-bromobut-2-ene (1.2 g, 6.78 mmol) in dimethylformamide (5 ml) was heated under reflux for 3 h. The mixture was then cooled and the volatiles were removed *in vacuo*. The residue was crystallized from a DMF/EtOH (1:3) mixture [Yield: 1.39 g, 78%. Mp. 479–481°K].

Analysis, calculated for C_25_H_32_N_4_OBr_2_: C53.19, H 5.67, N 9.93%; found: C 53.95, H 5.42, N 10.11%. ^1^H-NMR (DMSO-d_6_): δ (p.p.m.) 8.24 (s, 2-CH, 2H), 2.64 (t, bridge, 2H), 4.83 (t, bridge, 4H), 1.86 (d, methyl, 6H), 5.14 (d, methylene, 4H), 5.77 (m, =CH, 4H), 7.70–8.05 (m, Ar—H, 8H). ^13^C-NMR (DMSO-d_6_): δ (p.p.m.) 13.28, 17.56, 28.28, 43.70, 48.44, 113.74, 122.22, 123.37, 126.50, 130.89, 131.52, 132.89, 142.18.

### Refinement

The H atoms of the water molecule were located from a difference Fourier map and refined freely. The C-bound H atoms were located geometrically, with C—H = 0.93 - 0.97 Å, and refined using a riding model, with *U*_iso_(H) = 1.2*U*_eq_(C*_aromatic _*and *C_methylene_*) or 1.5*U*_eq_(*C_methyl_*). The  C atoms  of  both –CH_2_(CH)_2_CH_3_ groups in (I) show very high thermal parameters but a suitable disorder model could not be found to separate discrete disorder components

### Crystal data

**Table:**

C_25_H_30_N_4_^2+^·2Br^−^·H_2_O	*Z* = 2
*M**_r_* = 564.35	*F*(000) = 576
Triclinic, *P*1	*D*_x_ = 1.420 Mg m^−^^3^
Hall symbol: -P 1	Mo *K*α radiation, λ = 0.71073 Å
*a* = 8.7989 (9) Å	Cell parameters from 25470 reflections
*b* = 11.1878 (13) Å	θ = 2.0–28.0°
*c* = 14.8813 (14) Å	µ = 3.09 mm^−^^1^
α = 106.381 (8)°	*T* = 296 K
β = 96.490 (8)°	Prismatic plate, light yellow
γ = 106.227 (8)°	0.53 × 0.45 × 0.31 mm
*V* = 1319.9 (3) Å^3^	

### Data collection

**Table:**

Stoe IPDSII diffractometer	6181 independent reflections
Radiation source: sealed X-ray tube, 12 x 0.4 mm long-fine focus	4101 reflections with *I* > 2σ(*I*)
plane graphite	*R*_int_ = 0.063
Detector resolution: 6.67 pixels mm^-1^	θ_max_ = 27.7°, θ_min_ = 2.5°
ω scans	*h* = −11→11
Absorption correction: integration (X-RED32; Stoe & Cie, 2002)	*k* = −14→13
*T*_min_ = 0.291, *T*_max_ = 0.447	*l* = −18→19
16109 measured reflections	

### Refinement

**Table:**

Refinement on *F*^2^	Primary atom site location: structure-invariant direct methods
Least-squares matrix: full	Secondary atom site location: difference Fourier map
*R*[*F*^2^ > 2σ(*F*^2^)] = 0.054	Hydrogen site location: inferred from neighbouring sites
*wR*(*F*^2^) = 0.124	H atoms treated by a mixture of independent and constrained refinement
*S* = 1.05	*w* = 1/[σ^2^(*F*_o_^2^) + (0.0416*P*)^2^ + 0.9807*P*] where *P* = (*F*_o_^2^ + 2*F*_c_^2^)/3
6181 reflections	(Δ/σ)_max_ < 0.001
297 parameters	Δρ_max_ = 0.56 e Å^−^^3^
0 restraints	Δρ_min_ = −0.41 e Å^−^^3^

### Special details

**Table:**

Geometry. Bond distances, angles *etc*. have been calculated using the rounded fractional coordinates. All su's are estimated from the variances of the (full) variance-covariance matrix. The cell e.s.d.'s are taken into account in the estimation of distances, angles and torsion angles
Refinement. Refinement on *F*^2^ for ALL reflections except those flagged by the user for potential systematic errors. Weighted *R*-factors *wR* and all goodnesses of fit *S* are based on *F*^2^, conventional *R*-factors *R* are based on *F*, with *F* set to zero for negative *F*^2^. The observed criterion of *F*^2^ > σ(*F*^2^) is used only for calculating -*R*-factor-obs *etc*. and is not relevant to the choice of reflections for refinement. *R*-factors based on *F*^2^ are statistically about twice as large as those based on *F*, and *R*-factors based on ALL data will be even larger.

### Fractional atomic coordinates and isotropic or equivalent isotropic displacement parameters (Å^2^)

**Table:**

	*x*	*y*	*z*	*U*_iso_*/*U*_eq_	
N1	0.7390 (4)	0.6064 (4)	0.4072 (2)	0.0525 (10)	
N2	0.4931 (4)	0.5116 (3)	0.3227 (2)	0.0495 (10)	
N3	0.1559 (4)	0.3113 (3)	0.0400 (2)	0.0463 (10)	
N4	0.2504 (4)	0.1820 (4)	−0.0600 (2)	0.0557 (11)	
C1	1.1336 (19)	0.851 (2)	0.3031 (16)	0.400 (19)	
C2	1.0601 (14)	0.774 (2)	0.3445 (13)	0.292 (12)	
C3	0.9949 (10)	0.7489 (12)	0.4033 (10)	0.177 (6)	
C4	0.9166 (5)	0.6413 (6)	0.4322 (3)	0.0707 (18)	
C5	0.6392 (5)	0.6485 (4)	0.4651 (3)	0.0485 (12)	
C6	0.6724 (5)	0.7321 (4)	0.5592 (3)	0.0572 (14)	
C7	0.5407 (6)	0.7523 (5)	0.5946 (3)	0.0661 (16)	
C8	0.3830 (6)	0.6909 (5)	0.5413 (3)	0.0673 (17)	
C9	0.3504 (5)	0.6076 (4)	0.4486 (3)	0.0559 (14)	
C10	0.4821 (5)	0.5886 (4)	0.4116 (2)	0.0451 (11)	
C11	0.6481 (5)	0.5246 (4)	0.3234 (3)	0.0546 (14)	
C12	0.3576 (5)	0.4279 (4)	0.2430 (3)	0.0524 (12)	
C13	0.3047 (5)	0.5057 (4)	0.1841 (3)	0.0536 (12)	
C14	0.1475 (5)	0.4283 (4)	0.1120 (3)	0.0547 (14)	
C15	0.0246 (5)	0.2004 (4)	−0.0130 (2)	0.0462 (11)	
C16	−0.1379 (5)	0.1681 (5)	−0.0101 (3)	0.0607 (16)	
C17	−0.2356 (6)	0.0474 (5)	−0.0719 (4)	0.0730 (17)	
C18	−0.1753 (7)	−0.0361 (5)	−0.1343 (4)	0.0756 (17)	
C19	−0.0147 (6)	−0.0054 (5)	−0.1380 (3)	0.0692 (18)	
C20	0.0849 (5)	0.1169 (4)	−0.0768 (3)	0.0528 (14)	
C21	0.2866 (5)	0.2959 (4)	0.0079 (3)	0.0520 (14)	
C22	0.3684 (7)	0.1338 (7)	−0.1103 (3)	0.084 (2)	
C23	0.3596 (8)	0.1486 (7)	−0.2051 (4)	0.094 (3)	
C24	0.3622 (10)	0.0582 (8)	−0.2808 (5)	0.116 (3)	
C25	0.3606 (14)	0.0664 (10)	−0.3775 (6)	0.162 (5)	
O1	0.5845 (5)	0.2069 (5)	0.1612 (4)	0.0878 (16)	
Br1	0.70894 (5)	0.46584 (5)	0.08608 (3)	0.0654 (2)	
Br2	−0.05435 (5)	0.30445 (5)	0.30568 (3)	0.0648 (2)	
H1A	1.16160	0.80090	0.24750	0.5960*	
H1B	1.23030	0.91410	0.34640	0.5960*	
H1C	1.06430	0.89630	0.28420	0.5960*	
H2	1.05770	0.69140	0.30580	0.3530*	
H3	0.99330	0.82660	0.44670	0.2120*	
H4A	0.95090	0.66410	0.50090	0.0840*	
H4B	0.94770	0.56570	0.40120	0.0840*	
H6	0.77720	0.77180	0.59580	0.0680*	
H7	0.55760	0.80940	0.65670	0.0800*	
H8	0.29780	0.70650	0.56900	0.0810*	
H9	0.24520	0.56620	0.41270	0.0670*	
H11	0.68710	0.48220	0.27240	0.0660*	
H12A	0.26710	0.38660	0.26790	0.0630*	
H12B	0.38910	0.35850	0.20210	0.0630*	
H13A	0.29250	0.58410	0.22700	0.0650*	
H13B	0.38890	0.53390	0.15050	0.0650*	
H14A	0.06290	0.40110	0.14570	0.0660*	
H14B	0.11790	0.48530	0.07980	0.0660*	
H16	−0.17850	0.22470	0.03130	0.0730*	
H17	−0.34570	0.02090	−0.07200	0.0870*	
H18	−0.24680	−0.11630	−0.17550	0.0910*	
H19	0.02510	−0.06320	−0.17900	0.0830*	
H21	0.38980	0.35770	0.03050	0.0620*	
H22A	0.47680	0.18260	−0.07220	0.1010*	
H22B	0.34740	0.04150	−0.11690	0.1010*	
H23	0.35180	0.22730	−0.21120	0.1130*	
H24	0.36530	−0.02080	−0.27320	0.1390*	
H25A	0.36370	−0.01500	−0.41980	0.2430*	
H25B	0.45340	0.13740	−0.37610	0.2430*	
H25C	0.26380	0.08230	−0.39980	0.2430*	
HW1	0.625 (9)	0.268 (7)	0.143 (5)	0.11 (3)*	
HW2	0.677 (10)	0.224 (7)	0.201 (5)	0.12 (3)*	

### Atomic displacement parameters (Å^2^)

**Table:**

	*U*^11^	*U*^22^	*U*^33^	*U*^12^	*U*^13^	*U*^23^
N1	0.0407 (17)	0.070 (2)	0.0457 (17)	0.0218 (17)	0.0100 (13)	0.0135 (16)
N2	0.0431 (18)	0.058 (2)	0.0410 (16)	0.0144 (15)	0.0074 (13)	0.0091 (14)
N3	0.0391 (16)	0.055 (2)	0.0418 (16)	0.0164 (15)	0.0081 (12)	0.0107 (14)
N4	0.052 (2)	0.074 (2)	0.0436 (17)	0.0306 (18)	0.0127 (14)	0.0120 (17)
C1	0.177 (14)	0.60 (4)	0.59 (4)	0.059 (18)	0.041 (18)	0.53 (3)
C2	0.089 (8)	0.50 (3)	0.40 (2)	0.047 (12)	0.045 (10)	0.37 (2)
C3	0.057 (5)	0.233 (12)	0.288 (14)	0.033 (6)	0.035 (6)	0.173 (11)
C4	0.043 (2)	0.108 (4)	0.061 (3)	0.032 (3)	0.0097 (19)	0.021 (3)
C5	0.044 (2)	0.058 (2)	0.045 (2)	0.0184 (19)	0.0102 (16)	0.0173 (18)
C6	0.055 (2)	0.068 (3)	0.043 (2)	0.024 (2)	0.0019 (17)	0.0093 (19)
C7	0.074 (3)	0.079 (3)	0.046 (2)	0.037 (3)	0.014 (2)	0.009 (2)
C8	0.063 (3)	0.087 (3)	0.057 (3)	0.038 (3)	0.023 (2)	0.014 (2)
C9	0.046 (2)	0.067 (3)	0.057 (2)	0.021 (2)	0.0142 (18)	0.020 (2)
C10	0.045 (2)	0.048 (2)	0.0401 (18)	0.0142 (17)	0.0084 (15)	0.0125 (16)
C11	0.046 (2)	0.069 (3)	0.043 (2)	0.018 (2)	0.0134 (16)	0.0088 (19)
C12	0.046 (2)	0.050 (2)	0.047 (2)	0.0062 (18)	0.0038 (16)	0.0061 (17)
C13	0.052 (2)	0.054 (2)	0.046 (2)	0.0144 (19)	0.0048 (17)	0.0080 (18)
C14	0.051 (2)	0.063 (3)	0.046 (2)	0.024 (2)	0.0077 (17)	0.0073 (18)
C15	0.046 (2)	0.053 (2)	0.0389 (18)	0.0184 (18)	0.0054 (15)	0.0133 (16)
C16	0.042 (2)	0.073 (3)	0.065 (3)	0.020 (2)	0.0046 (19)	0.021 (2)
C17	0.046 (3)	0.076 (3)	0.088 (3)	0.011 (2)	−0.003 (2)	0.029 (3)
C18	0.072 (3)	0.059 (3)	0.071 (3)	0.006 (3)	−0.021 (2)	0.013 (2)
C19	0.080 (4)	0.062 (3)	0.055 (2)	0.031 (3)	−0.005 (2)	0.003 (2)
C20	0.052 (2)	0.063 (3)	0.043 (2)	0.023 (2)	0.0033 (17)	0.0151 (18)
C21	0.040 (2)	0.068 (3)	0.043 (2)	0.0157 (19)	0.0085 (15)	0.0129 (19)
C22	0.073 (3)	0.117 (5)	0.063 (3)	0.051 (3)	0.025 (2)	0.007 (3)
C23	0.100 (5)	0.096 (4)	0.080 (4)	0.030 (4)	0.044 (3)	0.013 (3)
C24	0.159 (7)	0.124 (6)	0.082 (4)	0.058 (5)	0.042 (4)	0.042 (4)
C25	0.221 (11)	0.175 (9)	0.114 (6)	0.072 (8)	0.071 (7)	0.061 (6)
O1	0.054 (2)	0.091 (3)	0.103 (3)	0.006 (2)	0.007 (2)	0.030 (2)
Br1	0.0531 (3)	0.0932 (4)	0.0649 (3)	0.0309 (3)	0.0197 (2)	0.0390 (3)
Br2	0.0507 (3)	0.0840 (4)	0.0557 (3)	0.0243 (2)	0.0134 (2)	0.0140 (2)

### Geometric parameters (Å, °)

**Table:**

O1—HW2	0.89 (9)	C23—C24	1.292 (10)
O1—HW1	0.81 (8)	C24—C25	1.466 (12)
N1—C11	1.324 (5)	C1—H1C	0.9600
N1—C4	1.476 (6)	C1—H1A	0.9600
N1—C5	1.382 (6)	C1—H1B	0.9600
N2—C11	1.329 (6)	C2—H2	0.9300
N2—C12	1.461 (5)	C3—H3	0.9300
N2—C10	1.391 (4)	C4—H4A	0.9700
N3—C21	1.330 (6)	C4—H4B	0.9700
N3—C14	1.466 (5)	C6—H6	0.9300
N3—C15	1.392 (5)	C7—H7	0.9300
N4—C22	1.480 (7)	C8—H8	0.9300
N4—C20	1.391 (6)	C9—H9	0.9300
N4—C21	1.312 (6)	C11—H11	0.9300
C1—C2	1.27 (3)	C12—H12A	0.9700
C2—C3	1.16 (2)	C12—H12B	0.9700
C3—C4	1.416 (15)	C13—H13A	0.9700
C5—C6	1.392 (6)	C13—H13B	0.9700
C5—C10	1.388 (6)	C14—H14A	0.9700
C6—C7	1.374 (7)	C14—H14B	0.9700
C7—C8	1.392 (7)	C16—H16	0.9300
C8—C9	1.375 (6)	C17—H17	0.9300
C9—C10	1.381 (6)	C18—H18	0.9300
C12—C13	1.519 (6)	C19—H19	0.9300
C13—C14	1.512 (6)	C21—H21	0.9300
C15—C16	1.382 (7)	C22—H22B	0.9700
C15—C20	1.395 (6)	C22—H22A	0.9700
C16—C17	1.371 (8)	C23—H23	0.9300
C17—C18	1.384 (8)	C24—H24	0.9300
C18—C19	1.369 (8)	C25—H25C	0.9600
C19—C20	1.384 (7)	C25—H25A	0.9600
C22—C23	1.462 (8)	C25—H25B	0.9600
			
Br1···C21	3.550 (5)	C19···H1C^i^	2.8300
Br1···O1	3.342 (6)	C21···H12B	2.7500
Br1···C11	3.536 (4)	C21···H13B	2.7300
Br1···C14^i^	3.696 (4)	C22···H19	3.0600
Br1···C21^i^	3.335 (5)	HW1···H11	2.4900
Br1···N3^i^	3.555 (3)	HW1···Br1	2.54 (8)
Br2···O1^ii^	3.333 (5)	HW1···H16^iii^	2.5800
Br1···HW1	2.54 (8)	H1C···C19^i^	2.8300
Br1···H11	2.7600	H2···H4B	2.3700
Br1···H21	2.6500	HW2···Br2^iii^	2.45 (8)
Br1···H16^iii^	3.0600	H4A···H6	2.5200
Br1···H14B^i^	3.1400	H4A···C6	2.9100
Br1···H21^i^	3.2000	H4A···Br2^iv^	2.8100
Br2···H14A	3.0500	H4B···H2	2.3700
Br2···HW2^ii^	2.45 (8)	H4B···H11	2.5700
Br2···H4B^ii^	2.8600	H4B···Br2^iii^	2.8600
Br2···H4A^iv^	2.8100	H6···Br2^iv^	3.1400
Br2···H6^iv^	3.1400	H6···C4	3.0400
Br2···H8^v^	2.9900	H6···H4A	2.5200
Br2···H19^vi^	2.9200	H8···Br2^v^	2.9900
Br2···H9	3.1900	H9···H12A	2.5700
Br2···H12A	2.8900	H9···C12	2.9900
O1···Br2^iii^	3.333 (5)	H9···Br2	3.1900
O1···C17^vi^	3.369 (8)	H11···Br1	2.7600
O1···Br1	3.342 (6)	H11···H4B	2.5700
O1···H12B	2.7400	H11···O1	2.8800
O1···H22B^vii^	2.9200	H11···HW1	2.4900
O1···H11	2.8800	H11···H12B	2.5300
O1···H18^vi^	2.9100	H12A···C9	2.9400
O1···H17^vi^	2.6700	H12A···H9	2.5700
N1···N2	2.177 (5)	H12A···Br2	2.8900
N2···N1	2.177 (5)	H12A···H14A	2.4800
N3···Br1^i^	3.555 (3)	H12B···N3	2.8000
N3···N4	2.176 (5)	H12B···O1	2.7400
N4···N3	2.176 (5)	H12B···H18^vi^	2.5400
N3···H12B	2.8000	H12B···H21	2.5500
C1···C19^i^	3.60 (2)	H12B···H11	2.5300
C5···C9^iv^	3.473 (6)	H12B···C21	2.7500
C8···C11^iv^	3.526 (7)	H13A···C10	3.0300
C9···C5^iv^	3.473 (6)	H13B···H21	2.2600
C11···C8^iv^	3.526 (7)	H13B···C21	2.7300
C11···Br1	3.536 (4)	H14A···Br2	3.0500
C12···C21	3.300 (6)	H14A···C16	2.9000
C14···Br1^i^	3.696 (4)	H14A···H12A	2.4800
C15···C18^vi^	3.594 (7)	H14A···H16	2.5000
C15···C19^vi^	3.538 (6)	H14B···Br1^i^	3.1400
C16···C19^vi^	3.596 (7)	H16···C14	2.9800
C17···O1^vi^	3.369 (8)	H16···Br1^ii^	3.0600
C18···C20^vi^	3.572 (7)	H16···HW1^ii^	2.5800
C18···C15^vi^	3.594 (7)	H16···H14A	2.5000
C19···C16^vi^	3.596 (7)	H17···O1^vi^	2.6700
C19···C15^vi^	3.538 (6)	H18···H12B^vi^	2.5400
C19···C1^i^	3.60 (2)	H18···O1^vi^	2.9100
C20···C18^vi^	3.572 (7)	H19···C22	3.0600
C21···Br1	3.550 (5)	H19···Br2^vi^	2.9200
C21···C12	3.300 (6)	H21···Br1	2.6500
C21···Br1^i^	3.335 (5)	H21···C13	2.7300
C4···H6	3.0400	H21···H12B	2.5500
C6···H4A	2.9100	H21···H13B	2.2600
C9···H12A	2.9400	H21···H22A	2.4800
C10···H13A	3.0300	H21···Br1^i^	3.2000
C12···H9	2.9900	H22A···H21	2.4800
C13···H21	2.7300	H22B···C19	3.0400
C14···H16	2.9800	H22B···H24	2.2700
C16···H14A	2.9000	H22B···O1^vii^	2.9200
C19···H22B	3.0400	H24···H22B	2.2700
			
HW1—O1—HW2	91 (7)	N1—C4—H4B	109.00
C4—N1—C11	124.1 (4)	C3—C4—H4B	109.00
C5—N1—C11	108.5 (4)	H4A—C4—H4B	108.00
C4—N1—C5	127.4 (3)	C3—C4—H4A	109.00
C10—N2—C11	108.2 (3)	C5—C6—H6	122.00
C11—N2—C12	125.7 (3)	C7—C6—H6	122.00
C10—N2—C12	126.1 (4)	C6—C7—H7	119.00
C14—N3—C15	125.6 (4)	C8—C7—H7	119.00
C15—N3—C21	107.6 (3)	C9—C8—H8	119.00
C14—N3—C21	126.6 (4)	C7—C8—H8	119.00
C20—N4—C21	108.8 (4)	C10—C9—H9	122.00
C20—N4—C22	126.5 (4)	C8—C9—H9	122.00
C21—N4—C22	124.7 (4)	N1—C11—H11	125.00
C1—C2—C3	155 (2)	N2—C11—H11	125.00
C2—C3—C4	142.0 (16)	N2—C12—H12A	109.00
N1—C4—C3	111.2 (6)	N2—C12—H12B	109.00
N1—C5—C10	106.8 (3)	C13—C12—H12B	109.00
C6—C5—C10	121.5 (4)	H12A—C12—H12B	108.00
N1—C5—C6	131.7 (4)	C13—C12—H12A	109.00
C5—C6—C7	115.8 (4)	C12—C13—H13A	109.00
C6—C7—C8	122.6 (4)	C14—C13—H13A	109.00
C7—C8—C9	121.4 (5)	C14—C13—H13B	109.00
C8—C9—C10	116.4 (4)	C12—C13—H13B	109.00
N2—C10—C5	106.3 (4)	H13A—C13—H13B	108.00
C5—C10—C9	122.1 (3)	N3—C14—H14B	109.00
N2—C10—C9	131.6 (4)	C13—C14—H14A	109.00
N1—C11—N2	110.2 (4)	C13—C14—H14B	109.00
N2—C12—C13	111.9 (4)	H14A—C14—H14B	108.00
C12—C13—C14	113.4 (4)	N3—C14—H14A	109.00
N3—C14—C13	113.3 (4)	C17—C16—H16	122.00
N3—C15—C16	131.2 (4)	C15—C16—H16	122.00
C16—C15—C20	122.0 (4)	C16—C17—H17	119.00
N3—C15—C20	106.8 (4)	C18—C17—H17	119.00
C15—C16—C17	116.0 (4)	C19—C18—H18	119.00
C16—C17—C18	121.9 (5)	C17—C18—H18	119.00
C17—C18—C19	122.7 (5)	C18—C19—H19	122.00
C18—C19—C20	115.9 (5)	C20—C19—H19	122.00
N4—C20—C15	105.8 (4)	N3—C21—H21	125.00
N4—C20—C19	132.7 (4)	N4—C21—H21	125.00
C15—C20—C19	121.4 (4)	N4—C22—H22B	109.00
N3—C21—N4	110.9 (4)	C23—C22—H22A	109.00
N4—C22—C23	111.9 (5)	C23—C22—H22B	109.00
C22—C23—C24	123.5 (7)	H22A—C22—H22B	108.00
C23—C24—C25	127.2 (9)	N4—C22—H22A	109.00
C2—C1—H1A	110.00	C24—C23—H23	118.00
C2—C1—H1B	109.00	C22—C23—H23	118.00
H1A—C1—H1B	109.00	C23—C24—H24	116.00
H1A—C1—H1C	109.00	C25—C24—H24	116.00
H1B—C1—H1C	109.00	C24—C25—H25B	109.00
C2—C1—H1C	109.00	C24—C25—H25C	110.00
C3—C2—H2	103.00	C24—C25—H25A	109.00
C1—C2—H2	103.00	H25A—C25—H25C	110.00
C2—C3—H3	109.00	H25B—C25—H25C	109.00
C4—C3—H3	109.00	H25A—C25—H25B	110.00
N1—C4—H4A	109.00		
			
C11—N1—C5—C10	−0.5 (5)	C22—N4—C20—C19	2.6 (8)
C4—N1—C11—N2	179.3 (4)	C22—N4—C20—C15	−179.7 (4)
C5—N1—C4—C3	−94.8 (8)	C1—C2—C3—C4	179 (3)
C11—N1—C4—C3	86.9 (8)	C2—C3—C4—N1	−102.2 (19)
C4—N1—C5—C6	0.2 (8)	C6—C5—C10—C9	−0.5 (7)
C11—N1—C5—C6	178.8 (5)	C6—C5—C10—N2	−179.2 (4)
C4—N1—C5—C10	−179.1 (5)	C10—C5—C6—C7	−0.8 (7)
C5—N1—C11—N2	0.7 (5)	N1—C5—C6—C7	−180.0 (5)
C11—N2—C10—C9	−178.3 (5)	N1—C5—C10—C9	178.9 (4)
C12—N2—C10—C9	0.1 (7)	N1—C5—C10—N2	0.2 (5)
C10—N2—C11—N1	−0.5 (5)	C5—C6—C7—C8	1.6 (7)
C12—N2—C11—N1	−179.0 (4)	C6—C7—C8—C9	−1.2 (8)
C10—N2—C12—C13	83.4 (5)	C7—C8—C9—C10	−0.1 (7)
C11—N2—C12—C13	−98.4 (5)	C8—C9—C10—C5	0.9 (7)
C12—N2—C10—C5	178.7 (4)	C8—C9—C10—N2	179.3 (5)
C11—N2—C10—C5	0.2 (5)	N2—C12—C13—C14	−169.9 (4)
C21—N3—C14—C13	−29.1 (6)	C12—C13—C14—N3	−62.0 (5)
C15—N3—C14—C13	156.7 (4)	N3—C15—C20—N4	−0.6 (4)
C14—N3—C15—C16	−3.4 (7)	N3—C15—C16—C17	−178.5 (4)
C15—N3—C21—N4	−1.7 (5)	C20—C15—C16—C17	1.7 (7)
C14—N3—C21—N4	−176.7 (4)	C16—C15—C20—C19	−2.7 (7)
C14—N3—C15—C20	176.4 (4)	N3—C15—C20—C19	177.6 (4)
C21—N3—C15—C20	1.4 (4)	C16—C15—C20—N4	179.3 (4)
C21—N3—C15—C16	−178.4 (5)	C15—C16—C17—C18	−0.9 (8)
C21—N4—C20—C15	−0.4 (5)	C16—C17—C18—C19	0.9 (9)
C22—N4—C21—N3	−179.4 (4)	C17—C18—C19—C20	−1.6 (8)
C20—N4—C22—C23	77.2 (7)	C18—C19—C20—N4	180.0 (5)
C20—N4—C21—N3	1.3 (5)	C18—C19—C20—C15	2.5 (7)
C21—N4—C22—C23	−101.9 (6)	N4—C22—C23—C24	−136.1 (8)
C21—N4—C20—C19	−178.2 (5)	C22—C23—C24—C25	−177.3 (9)

Symmetry codes: (i) −*x*+1, −*y*+1, −*z*; (ii) *x*−1, *y*, *z*; (iii) *x*+1, *y*, *z*; (iv) −*x*+1, −*y*+1, −*z*+1; (v) −*x*, −*y*+1, −*z*+1; (vi) −*x*, −*y*, −*z*; (vii) −*x*+1, −*y*, −*z*.

### Hydrogen-bond geometry (Å, °)

**Table:**

*D*—H···*A*	*D*—H	H···*A*	*D*···*A*	*D*—H···*A*
O1—HW1···Br1	0.81 (8)	2.54 (8)	3.342 (6)	171 (8)
O1—HW2···Br2^iii^	0.89 (9)	2.45 (8)	3.333 (5)	171 (7)
C4—H4A···Br2^iv^	0.97	2.81	3.773 (4)	172
C4—H4B···Br2^iii^	0.97	2.86	3.802 (6)	164
C11—H11···Br1	0.93	2.76	3.536 (4)	142
C12—H12A···Br2	0.97	2.89	3.822 (5)	161
C19—H19···Br2^vi^	0.93	2.92	3.816 (5)	162
C21—H21···Br1	0.93	2.65	3.550 (5)	162

Symmetry codes: (iii) *x*+1, *y*, *z*; (iv) −*x*+1, −*y*+1, −*z*+1; (vi) −*x*, −*y*, −*z*.

## References

[bb1] Akkurt, M., Öztürk, S., Küçükbay, H., Okuyucu, N. & Fun, H.-K. (2003). *Acta Cryst.* E**59**, o786–o788.

[bb2] Akkurt, M., Yıldırım, S. Ö., Küçükbay, H., Şireci, N. & Büyükgüngör, O. (2006). *Acta Cryst.* E**62**, o3184–o3186.

[bb3] Allen, F. H., Kennard, O., Watson, D. G., Brammer, L., Orpen, A. G. & Taylor, R. (1987). *J. Chem. Soc. Perkin Trans. 2*, pp. S1–19.

[bb4] Altomare, A., Burla, M. C., Camalli, M., Cascarano, G. L., Giacovazzo, C., Guagliardi, A., Moliterni, A. G. G., Polidori, G. & Spagna, R. (1999). *J. Appl. Cryst.* **32**, 115–119.

[bb5] Farrugia, L. J. (1997). *J. Appl. Cryst.* **30**, 565.

[bb6] Farrugia, L. J. (1999). *J. Appl. Cryst.* **32**, 837–838.

[bb7] Hall, J. E., Kerrigan, J. E., Ramachandran, K., Bender, B. C., Stanko, J. P., Jones, S. K., Patric, D. A. & Tidwell, R. R. (1998). *Antimicrob. Agents Chemother.* **42**, 666–674. 10.1128/aac.42.3.666 PMC105515 9517949

[bb8] Küçükbay, H., Çetinkaya, E. & Durmaz, R. (1995). *Arzneim. Forsch.* **45**, 1331–1334. 8595095

[bb9] Küçükbay, H., Durmaz, R., Güven, M. & Günal, S. (2001). *Arzneim. Forsch.* **51**, 420–424. 10.1055/s-0031-1300057 11413744

[bb10] Öztürk, S., Akkurt, M., Küçükbay, H., Okuyucu, N. & Fun, H.-K. (2003). *Acta Cryst.* E**59**, o1014–o1016.

[bb11] Sakai, T., Hamada, T., Awata, N. & Watanabe, J. (1989). *J. Pharmacobiodyn.* **12**, 530–536. 10.1248/bpb1978.12.530 2575663

[bb12] Sheldrick, G. M. (2008). *Acta Cryst.* A**64**, 112–122. 10.1107/S0108767307043930 18156677

[bb13] Stoe & Cie (2002). *X-AREA* and *X-RED32* Stoe & Cie, Darmstadt, Germany.

[bb14] Tidwell, R. R., Jones, S. K., Naiman, N. A., Berger, L. C., Brake, W. B., Dykstra, C. C. & Hall, J. E. (1993). *Antimicrob. Agents Chemother.* **37**, 1713–1716. 10.1128/aac.37.8.1713 PMC188050 8215291

[bb15] Turner, P. R. & Denny, W. A. (1996). *Mutat. Res.* **335**, 141–169. 10.1016/0027-5107(96)00027-9 8781582

